# Abnormal Arm Movements as the Presenting Sign of a Childhood Spinal Cord Tumor

**DOI:** 10.1177/08830738251353463

**Published:** 2025-07-23

**Authors:** Camiel A Wijngaarde, Elisabeth A Cats, A Fleur van Raamt, Eelco W Hoving, Kim Boshuisen

**Affiliations:** 1 Department of Neurology, 36512 Brain Center Rudolf Magnus, University Medical Center Utrecht, Utrecht, the Netherlands; 2 Department of Neurology, Gelre Hospital, Apeldoorn, the Netherlands; 3 Department of Radiology, Gelre Hospital, Apeldoorn, the Netherlands; 4 Department of Neuro-Oncology, Princess Máxima Center for Pediatric Oncology, Utrecht, the Netherlands

**Keywords:** Childhood Spinal Cord Tumor, Neurologic Examination, Radiculopathy

Neurologic localization, i.e. determining what part of the nervous system has been affected based on history taking and neurologic examination, is arguably the most important step in the diagnostic process for a neurologist. The resulting clinical diagnosis aids formulation of a differential diagnosis, guides adequate use of ancillary procedures, and contributes to preventing delay of diagnosis. In young children this process can be particularly challenging, as signs and symptoms can be more difficult to recognize.^1^ Here, we present a case that underlines this difficulty in a young infant.

## Case Presentation

A 15-month-old girl, born after an uneventful pregnancy and with normal neurologic development, presented with stereotype movements of her right arm since 3 months (Video 1). Parents described short-lasting moments of spontaneous twisting and turning of the right arm, which appeared to be uncomfortable. The frequency was not progressive and had been fluctuating between 3 and 20 times a day. Outside of these moments, there were no abnormalities or discomfort, and no asymmetry in her general movement pattern or hand dexterity.

Initial neurologic examination was normal, except for the short stereotype, possibly hemiballistic movements of the right arm without clear sings of pain/discomfort or dystonic posturing.

**Figure 1.**
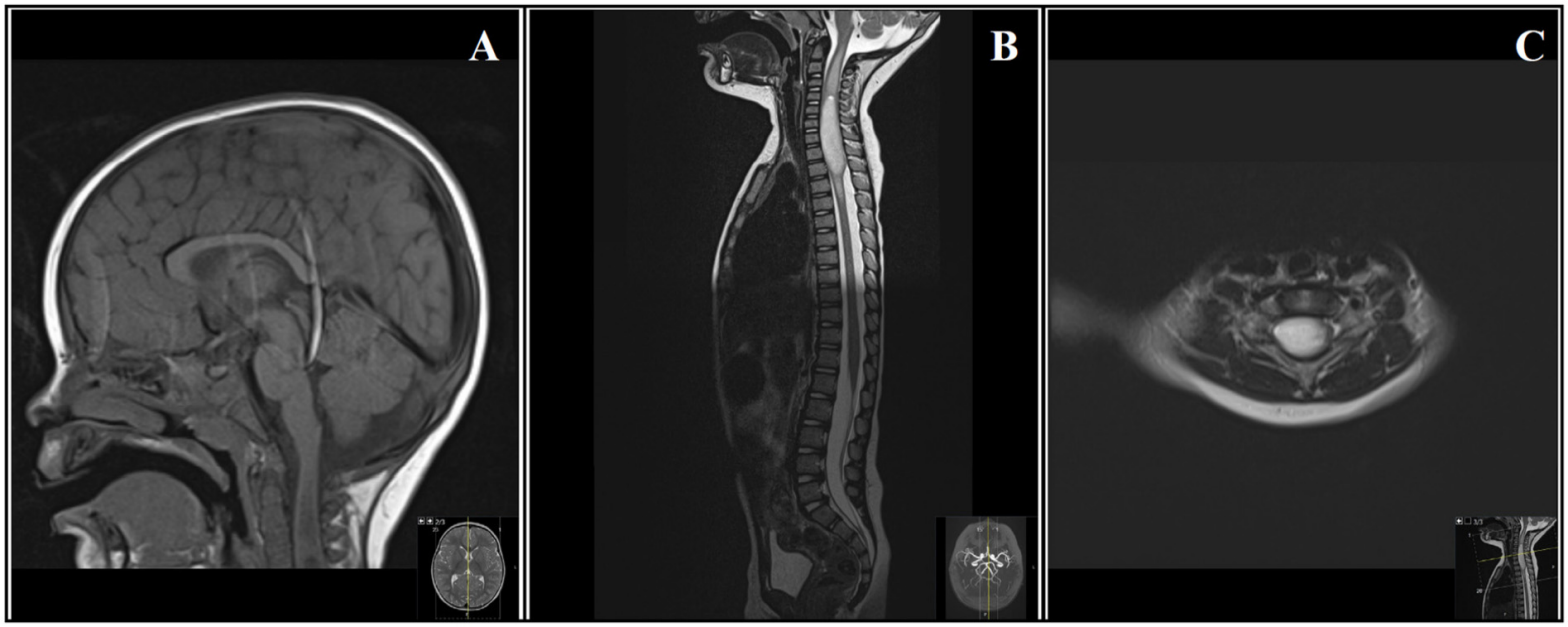
Magnetic Resonance Imaging. **(A**) Sagittal T1-weighted imaging of the brain, with the upper part of the cervical cord visible, showing a mass lesion. **(B**) Sagittal T2-weighted imaging of the spinal cord, showing an intramedullary mass in the cervicothoracic spinal cord (C5-Th3). **(C**) Axial T2-weighted imaging of the spinal cord, showing the compressive effect of the mass lesion, predominantly on the right C6 nerve root.

MRI/MRA showed no brain abnormalities, in particular no basal ganglia damage.^2^ However, as an incidental finding, a large intramedullary mass in the cervicothoracic spinal cord (C5-Th3), which also compressed the right C6 nerve root, was diagnosed (Figure 1).

## Discussion and Conclusions

Presenting symptoms of childhood spinal tumors can vary greatly. Back pain and focal neurological deficits, including paraparesis, are most prevalent but patients may also present with less specific symptoms like torticollis, delayed motor milestone achievement, early handedness, or weight loss.^3,4^ Ultimately most childhood spinal cord tumors are diagnosed with a significant delay, as they are rare, symptoms may wax and wane, and clinical presentations are often nonspecific.^4^

Our case highlights the difficulty in pattern recognition in young children. Initial symptoms were suspected to be hemiballistic and only at follow-up visits right-sided hyperreflexia was present, indicative of pyramidal tract involvement. Although some movement disorders are generated at the spinal level,^5^ we believe in our case the symptoms are ultimately best interpreted as a cervical radiculopathy, even though radicular pain is an atypical presenting symptom for childhood spinal tumors, not mentioned in larger case series.^3,4^

The child was referred to the national pediatric cancer center and underwent a cervicothoracic laminotomy (C4-Th3) and maximal safe resection of the intramedullary tumor mass under guidance of intraoperative neurophysiological monitoring. Ancillary tests demonstrated a pilocytic astrocytoma with a *KIAA1549-BRAF* fusion (WHO grade 1). The patient recovered well and the paroxysmal movements of the right arm resolved completely after surgery. At last follow-up, at the age of 4 years, there was a subtle right-sided hyperreflexia and a subtle circumduction gait of the right leg when running. She is attending primary school, including PE classes, and has learnt to ride her bike. Magnetic resonance imaging has remained stable over time, without signs of recurrent tumor growth.

## Supplemental Material

**Figure:** Video 1.
